# Eco-friendly electrochemical assay of oxytetracycline and flunixin in their veterinary injections and spiked milk samples

**DOI:** 10.1186/s13065-024-01282-4

**Published:** 2024-09-19

**Authors:** Yossra A. Trabik, Miriam F. Ayad, Amr M. Mahmoud, Hind A. Abdullatif, Adel M. Michael

**Affiliations:** 1 Pharmaceutical Analytical Chemistry Department, Faculty of Pharmacy, Ain Shams University, Cairo, Egypt; 2 Analytical Chemistry Department, Faculty of Pharmacy, Cairo University, Cairo, Egypt; 3 Pharmaceutical Chemistry Department, Faculty of Pharmacy, Ahram Canadian University, Cairo, Egypt

**Keywords:** Glassy carbon electrode, Solid-contact ion-selective electrodes, Flunixin meglumine, Oxytetracycline HCl, 2-Hydroxypropyl-β-cyclodextrin

## Abstract

Two solid-contact electrochemical sensors were developed for detection of each of oxytetracycline HCl (OXY), and the co-formulated non-steroidal anti-inflammatory drug flunixin meglumine (FLU) in veterinary formulations and animal-derived food products. The designed sensors were based on a glassy carbon electrode as the substrate material and high molecular weight polyvinyl chloride (PVC) polymeric ion-sensing membranes doped with multiwalled carbon nanotubes (MWCNTs) to improve the potential stability and minimize signal drift. For determination of OXY, the sensing membrane was modified with potassium tetrakis (4-chlorophenyl) borate (K-TCPB), which was employed as a cation exchanger, and 2-hydroxypropyl-β-cyclodextrin (HP-ßCD), which was used as an ionophore. A linear response within a concentration range of 1 × 10^− 6^-1 × 10^− 2^ M with a slope of 59.47 mV/decade over a pH range of 1–5 was recorded. For the first time, two potentiometric electrodes were developed for determination of FLU, where the sensing membrane was modified with tetra dodecyl ammonium chloride (TDDAC) as an anion exchanger. A linear response within a concentration range of 1 × 10^− 5^-1 × 10^− 2^ M and a slope of -58.21 mV/decade over a pH range of 6–11 was observed. The suggested sensors were utilized for the selective determination of each drug in pure powder form, in veterinary formulations, and in spiked milk samples, with mean recoveries ranging from 98.50 to 102.10, and without any observed interference. The results acquired by the proposed sensors were statistically analyzed and compared with those acquired by the official methods, and the results showed no significant difference.

**Graphical Abstract:**

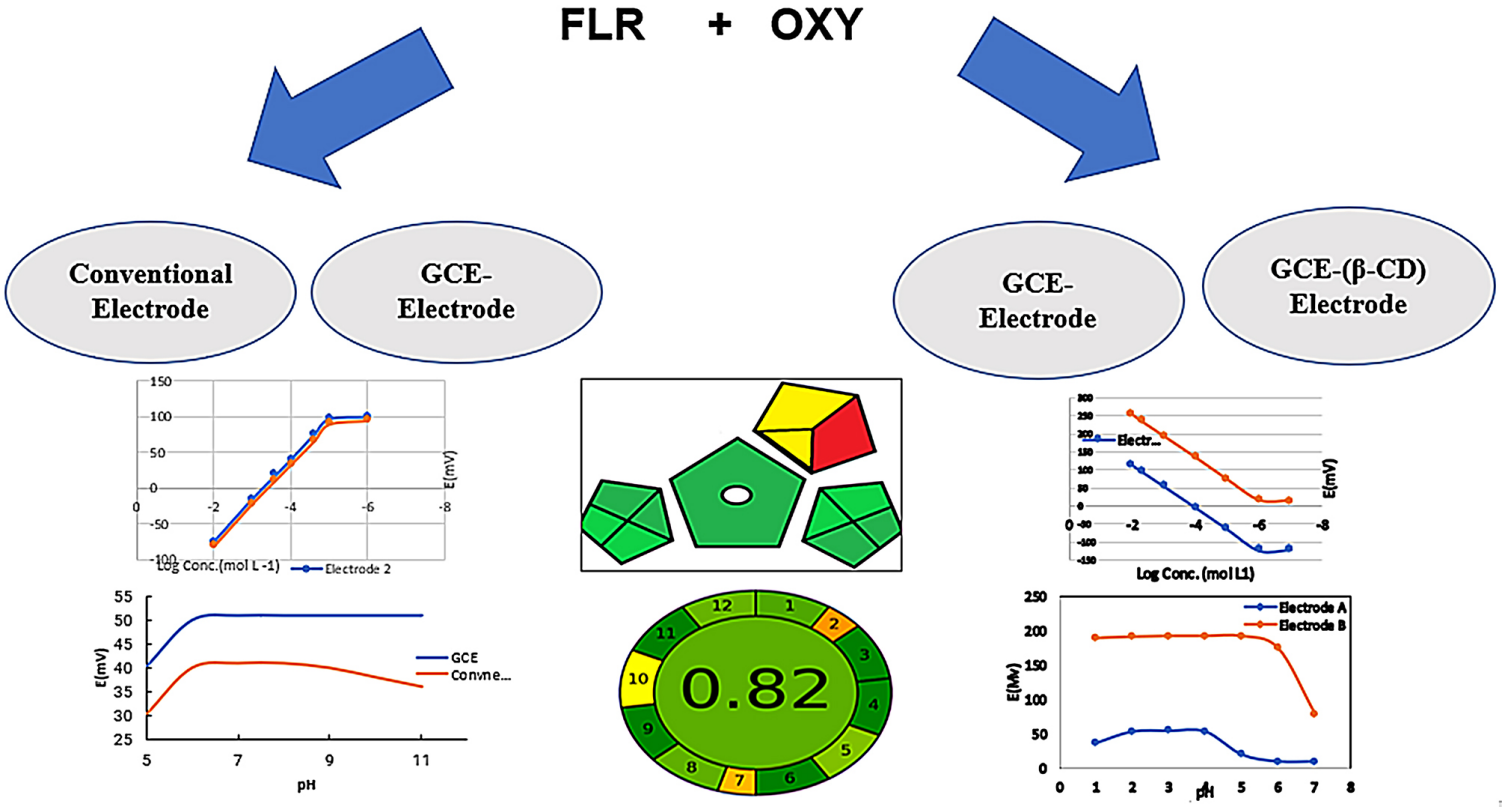

**Supplementary Information:**

The online version contains supplementary material available at 10.1186/s13065-024-01282-4.

## Introduction

Antimicrobial drugs are a mandatory part of human and veterinary medicine [1]. Unfortunately, as a result of excessive use and reliability, antimicrobial resistance (AMR) has become a very worrisome global issue [2]. In farming, antimicrobials, have been used particularly for treating bacterial infections as well as for growth promotion and prophylaxis [3]. It is common for farmers to treat entire livestock with antibiotics, although only a few of them are affected. Additionally, there are many cases in which antibiotics were described inappropriately in patients with viral infections. This massive use has been identified in the European Union (EU) as needless overuse. However, although antibiotics are used to promote growth, their preventive usage is still legal. Unfortunately, there is no clear regulatory mechanism controlling antibiotic contamination due to the lack of available information and methods for analyzing antibiotic residues in animal-derived food in Africa.

If antimicrobial residues are found in milk, production of microorganisms such as yogurt will be negatively affected. Moreover, very serious health issues, such as sensitivity and AMR, exist. Additionally, various temperatures have been shown to not affect the stability of various medicines, even after pasteurization processes reach 100 °C [4]. Traces of antibiotics were found in milk even after withdrawal periods [3].

According to previous reports, the most popular class of antibiotics sold in 2019 was tetracyclines [5]. oxytetracycline HCl (OXY) is a hydrophilic antibiotic, thus it tends to accumulate in aqueous water, which is the major component of milk. OXY is an antibiotic that is stable over a wide range of temperatures [4]. Additionally, tetracyclines are often removed from the body in their active form unchanged, and they accumulate and persist in the soil for a few months [3].

Knowing all this crucial and worrying information sets the aim of this study, which is to develop simple, accurate, environmentally friendly methods for the determination of OXY in milk, along with its combined drug, flunixin meglumine (FLU). Electrochemical methods offer the opportunity to apply green chemistry because of the safe solvents used, minimal to no sample pretreatment, and passive nature of these methods [6]. An ion selective electrode (ISE) is a widely used analytical approach for the determination of ion activity by measuring the electric potential. This method has multiple advantages over other techniques; it is easy to perform, has a relatively low cost, and offers a wide concentration range for measurement. Conventional liquid-contact ISEs were the first established ISEs and were based on internal solutions [7]. Unfortunately, these ISEs have multiple drawbacks, mainly including evaporation of the internal solution, a short life span, and unstable responses. Consequently, solid-state ISEs have been presented that satisfy all the demands of analytical ISEs, including long-term storage, stable reproducible responses, and easy handling.

It is well known that the materials of a sensor are the core elements needed to fabricate a high-performance electrochemical sensing electrode for selectively determining the target analyte. Nanoparticles have attracted increasing attention in the fabrication of stable solid contact ISEs (SC-ISE), that can be applied in various ways [8]. Fortunately, research combining nanotechnology and ISEs has opened wide doors to a variety of approaches. Conducting polymer nanoparticles [9, 10], gold nanoclusters [11], and carbon nanotubes [12] are examples of various approaches for fabricating sensors with reproducible calibration curves and superb stability.

Carbon nanotubes (CNTs) are molecular-scale wires with high chemical stability, high electrical conductivity, and outstanding mechanical strength [13]. Nanocomposite CNTs are dispersed on a variety of surfaces, making them perfect for SC-ISEs. CNTs are remarkably hydrophobic, so they interfere with the establishment of a water layer between the electrode and the sensing membrane. They also drastically decrease the signal drift, a disturbing disadvantage of SC-ISE [14]. Supramolecular macromolecules such as calixarenes and cyclodextrins have been widely used in the analytical field for targeted drug delivery, optical sensors and water pollution treatment. Cyclodextrins are used in various technologies, analytical methods, particularly in the fabrication of chemical sensors [15]. Because of their cage-like supramolecular structure, they are primarily included in host-guest chemical reactions in which covalent bonds are not developed with interacting radicals or ions. Cyclodextrins are well known for accommodating a vast range of molecules, forming inclusion complexes with drug or nanostructure supramolecular assemblies in their hydrophobic cavity [16]. Throughout its literature review, cyclodextrin has been used as an ionophore rendering potentiometric sensor to be highly durable and having excellent performance. These ionophores and nanocomposites (carbon nanotubes) may have synergistic effects that aid in the precise determination of the drugs studied.

**Fig. 1:**
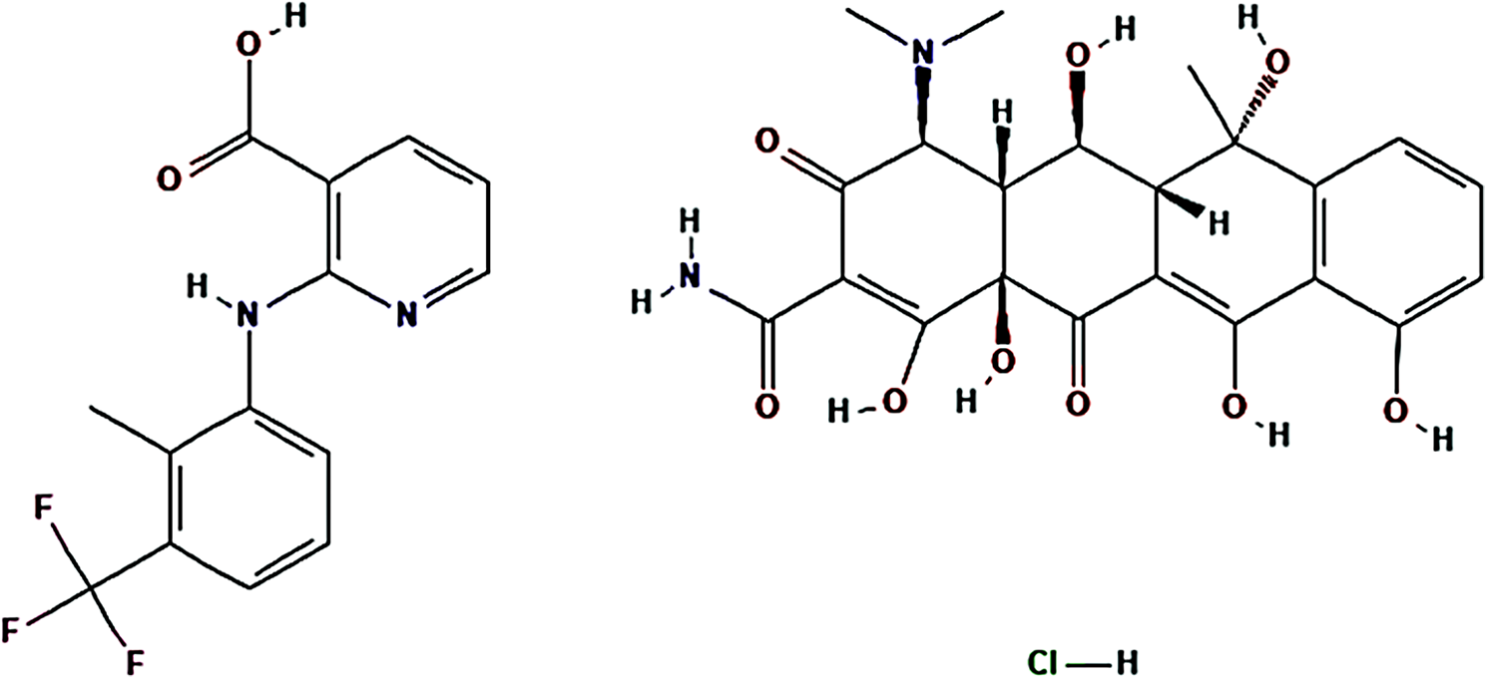
Chemical structure of FLU (**a**) and OXY (**b**)

Flunixin meglumine, designated (2R,3R,4R,5 S)-6-(methylamino) hexane-1,2,3,4,5-pentol;2-[2-methyl-3-(trifluoromethyl) anilino] pyridine-3-carboxylic acid (Fig. 1a), is a potent nonsteroidal analgesic utilized in veterinary medicine to treat large animals and a prostaglandin-endoperoxide synthase inhibitor [17].

Oxytetracycline hydrochloride, designated 4 S,4aR,5 S,5aR,6 S,12aR)-4-(dimethylamino)-1,5,6,10,11,12a-hexahydroxy-6-methyl-3,12-dioxo-4,4a,5,5a-tetrahydrotetracene-2-carboxamide; hydrochloride (Fig. 1b) is the oxytetracycline hydrochloride salt form. Oxytetracycline hydrochloride interferes with the binding of aminoacyl-tRNA to the mRNA-ribosome complex, thus inhibiting protein synthesis and preventing peptide elongation [17].

Literature reveals multiple analytical methods for quantifying FLU, including spectrophotometric [18], Surface enhanced Raman spectroscopy [20], HPLC [19, 22], LC-MS [23, 24], and voltametric [21, 25] methods. OXY was analyzed by spectrophotometry [26], HPLC [27, 28], and electrochemical [29] methods. Other methods reported simultaneous determination of FLU and OXY [30].

As far as we are aware, there is no reported method for the determination of FLU by ISEs. Considering OXY, no electrochemical methods using CNTs have been reported. In this work, three green techniques were utilized to fabricate conventional liquid contact ion selective electrode (LC-ISE) and SC-ISE devices by using a glass carbon electrode (GCE) -based potentiometric sensor and MWCNTs as an ion to electron transducer to determine both FLU and OXY. Methods were applied to veterinary injections and spiked milk samples. Considering FLU, both conventional and MWCNT-GCE were fabricated, and the results were compared. On the other hand, for OXY, the performance of the MWCNT-GCE utilizing cyclodextrin as an ionophore was comparable to that of a plain MWCNT-GCE. The developed methods were evaluated by both Green Analytical Procedure Index (GAPI) and Analytical GREEnness Metric Approach and Software (AGREE) greenness assessment tools.

## Experimental

### Instrument

**A** Jenway digital ion analyzer (model 3330; Essex, UK) was used, with a Ag/AgCl double-junction reference electrode (Thermo Scientific Orion 900200, USA; with 10% KNO_3_ as a bridge electrolyte and 3.0 M KCl with AgCl as an inner filling solution) (Aldrich Chemical Co. Steinheim, Germany).

### Materials and reagents

Flunixin meglumine (purity 100.08 ± 1.26) and oxytetracycline HCl (purity 101.08% ± 1.18) were supplied generously by Pharmswede Veterinary Company, (Cairo, Egypt). Floxon^®^ was obtained from Pharmswede Veterinary Company, (Cairo, Egypt).

Analytical grade boric acid, phosphoric acid, sodium hydroxide, and acetic acid were obtained from Piochem Company (Giza, Egypt). High molecular weight polyvinyl chloride (PVC), 2-hydroxypropyl-β-cyclodextrin (HP-ßCD), tetradodecylammonium chloride (TDDAC), and multiwalled carbon nanotubes (MWCNTs) were acquired from Fluka (Steinheim, Germany). Potassium tetrakis (4-chlorophenyl) borate (K-TCPB) and 2-nitrophenyl octyl ether (*o*-NPOE) were obtained from Sigma Aldrich (Steinheim, Germany). Tetrahydrofuran (THF) was acquired from BDH (Poole, England). Deionized water was obtained from SEDICO Pharmaceuticals Company, (Cairo, Egypt).

### Standard solutions

Standard stock solution (10^− 2^ M) was obtained by separately transferring 122.9 mg of FLU and 124.3 mg of OXY into 25-mL volumetric flasks and completing to the mark using Britton–Robinson buffer (BRB) at pH 8 and 3, respectively. A series of working solutions of lower concentrations were prepared by withdrawing different aliquots from each stock solution into two separate sets of 25-mL volumetric flasks and completing to the mark with BRB at pH 8 and 3, for FLU and OXY, respectively, to prepare a set of dilutions for each drug in concentration range of (10^− 7^–10^− 3^ M).

### Procedures

#### Preparation of the ion-selective membranes and electrodes assembly

##### Sensor fabrication for FLU determination

###### Ion-selective membranes for FLU

 For the first conventional electrode, *in* a Falcon tube, 95.0 mg of PVC, 5.0 mg of TDDAC and 0.2 mL of *o*-NPOE were mixed and subsequently dissolved in 3.0 mL of THF. The solution was then placed into a 5 cm Petri dish and covered with filter paper and left at room temperature overnight till completely dry. The resulting membrane was approximately 0.1 mm thick.

The second electrode (MWCNT-GCE) sensing cocktail was prepared by mashing 95.02% (95.51 mg) PVC with 4.97% (5.0 mg) TDDAC, dissolving the mixture in 0.2 mL of *o*-NPOE, and mixing the mixture with 3 mL of THF to obtain a homogenous mixture. Finally, 2 mL of MWCNTs working solution was added and mixed for ten minutes to obtain a uniform suspension.

###### Electrodes assembly

For the conventional electrode, from the master membrane, a disk of appropriate diameter was cut by a cork borer and then fixed with THF on the flat end of a PVC tubing, which was clipped to the end of the electrode glass.

A mixed solution consisting of the same volume of 10^− 2^ M FLU and 10^− 2^ M KCl was used as an internal reference solution. Ag/AgCl coated wire (1 mm diameter) was submerged in the internal reference solution as an internal reference electrode. The sensor was conditioned by placing it in a 10^− 2^ M solution of the drug for 24 h before use, after which the solution was used for electrode storage when not in use.

For MWCNT-GCE, 10 µL of the sensing suspension was dropped cast on a GCE and left for 24 h to dry. The sensor was soaked in darkness in 1.0 × 10^− 2^ M FLU for 24 h for appropriate conditioning and placed under the same conditions when left unused.

##### Sensor fabrication for OXY determination

###### Ion-selective membranes for OXY

For the ionophore-unmodified electrode (electrode A), *in* a Falcon tube, 33.17% (99.51 mg) PVC was mashed with 0.23% (0.69 mg) K-TCPB, dissolved in 66.6% (199.8 mg) o-NPOE, and mixed with 3 mL of THF until completely homogenous. Finally, 2 mL of MWCNTs working solution was added and mixed for 10 min to obtain a consistent suspension.

For the preparation of the ionophore-modified electrode (electrode B), 32.54% (97.62 mg) of PVC, 66.6% (199.8 mg) of o-NPOE, 0.23% (0.69 mg) of K-TCPB, and 0.63% (1.89 mg) of HP-ß CD ionophore were solubilized in 3 mL of THF until completely homogenous. Then, 2 mL of MWCNTs working solution was added, and the mixture was sonicated for 10 min to obtain a uniform suspension.

###### Electrodes assembly

Ten microliters of each of the two sensing cocktails were dropped cast on two separate GCEs and left for 24 h to dry. The sensors were soaked in darkness in 1.0 × 10^− 2^ M OXY for 24 h for conditioning purposes. The two electrodes were also placed under the same conditions when left unused.

#### Potentiometric measurements

The conditioned sensors were individually attached to a double junction Ag/AgCl reference electrode and calibrated afterwards by placing each electrode in its corresponding standard solutions while allowing it to calibrate during mixing. The electromotive forces were recorded once the potentiometer found a constant reading. After every single measurement, the sensors were washed with a buffer.

#### Study of the experimental conditions

##### Calibration of sensors

###### Identification of the slope, response time and operative life of the proposed sensors

The electrochemical performance of the proposed sensors was assessed according to the ICH guidelines [31]. The potentials obtained using the cited sensors were plotted against the log molar concentration of each matching drug. The slope was obtained from the linear fragment of the calibration graph. The detection limit was set at the intersection of the graph’s extrapolated linear segment. Moreover, the dynamic response of the electrodes was tested over the concentration range of 10^− 7^–10^− 2^ M for both drug solutions. Additionally, the life span of the sensor was investigated by periodical, repetitive observation of the slope of the drug calibration curve.

###### Effect of pH on the electrode response

The effect of pH on the potential of each electrode system was monitored over a pH range of 6–11 by immersing the electrode in a 10^− 3^ M solution of FLU.

For OXY, a 10^− 4^ M solution of OXY was observed over a pH range of 1–7. The different values were fixed using Britton–Robinson buffer, and the potential obtained at each pH was noted.

#### Application to veterinary formulation

OXY and FLU are found together in Floxon^®^ veterinary injection, which contains 33 mg/mL FLU, and 108 mg/ mL OXY. To determine FLU, 0.37 mL was taken from the injection and mixed to 25 mL with BR buffer (pH 8). To determine OXY, 0.12 mL was taken from the same dosage form, and the volume was completed to 25 mL with BR buffer (pH 3). The concentration resulting in both solutions was claimed to be 1 × 10^− 3^ M. Afterwards, the (EMF) produced by immersing each prepared electrode in combination with the reference electrode in the prepared solutions was recorded, and the corresponding regression equations were used to calculate the concentration of both drugs.

#### Application to spiked milk samples

Both 1 × 10^− 3^ M and 1 × 10^− 4^ M FLU and OXY were used. In a 25-mL volumetric flask, 12.5 mL of each concentration was added to the milk of animal origin (cow) and the final concentrations were 5 × 10^-4^ M and 5 × 10^− 5^ µg/mL, respectively. Subsequently, each electrode attached to the double-junction Ag/AgCl reference electrode was soaked in the prepared solutions. Finally, the resulting potentials were written down, and their concentrations were obtained from the corresponding equations.

#### Greenness assessment

The greenness of the developed methods was evaluated by the Green Analytical Procedure Index and the Analytical Greenness Metric Approach and Software.

## Results and discussion

Antibiotic residues found in animal-derived food have emerged as a pressing global issue [32]. Considering dairy farms, milk from cows that have administered antibiotics is considered unfit for human consumption. In addition, contamination of the milk bulk tank that contains these residues is a threat to consumption, and a loss to farmer financing. This study’s novelty is illustrated in attempting to establish a sensitive, fast, and reliable membrane electrode to selectively detect FLU, for the first time, by comparing the results of a conventional ISE with those of a SC-ISE [33] and OXY by comparing the results of an unmodified MWCNT-GCE with those of an ionophore-modified MWCNT-GCE, where MWCNTs are used for the first time in potentiometric determination of OXY, while abiding through the whole study by the Green Analytical Chemistry (GAC) rules.

Electrochemical sensors are characterized by exceptional analytical advantages in comparison to other conventional methods, which can be attributed to their special properties, including portability, ease of miniaturization, simplicity, low cost, great sensitivity and admirable selectivity [34].

### Ion-selective electrode performance

The potential generated emf is related to the activity (~ concentration at diluted solutions) of the analyte ion. The electrical potential (known as the phase boundary potential) is generated at the interface of the ion selective membrane (ISM) and sample solution by selective partitioning of ionic species between these two phases, resulting in interfacial charge separation.

The magnitude of the phase boundary potential depends on the activity of the ion in the membrane and the aqueous solution and governed by Nernst equation:

$$emf=E^{\circ}+(\text{R}T/zF)\:\text{Ln}\:a,$$

where an order of magnitude changes in the activity of an ion with charge z results in a 59.2 mV/z change in the potential of the ISE.

**Fig. 2:**
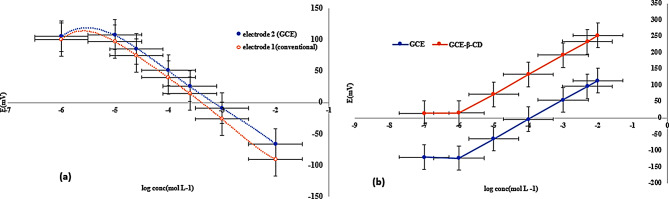
(**a**) Profile of the potential in mV VS log concentration of FLU in mol.L^− 1^ obtained by electrode 1(conventional) and electrode 2 (MWCNT-GCE), (**b**) Profile of the potential in mV VS log concentration of OXY in mol.L^− 1^ obtained by GCE electrode and GCE (HP-ßCD)electrode

Considering FLU, there are no ISEs reported in the literature for analyzing FLU, so there was a reason to start with the conventional ISE and compare it with SC-GCE. The latter electrode is based on coupling anionic FLU with the anionic exchanger TDDAC since the pKa of FLU is 5.80. As proven in the literature, there is a direct relationship between ion-exchanger lipophilicity and improvement in the detection limit, in addition to minimizing ion-exchanger leaching and hence improving the sensor lifetime. Anionic exchangers play a major role in the sensing performance of analytical sensors, where leakage outside the membrane limits the overall long-term stability of the sensors [35]. The anionic exchanger TDDAC was chosen because of its proven enhancement of selectivity and stability, which is attributed to the lipophilicity of TDDAC [35]. Thus, the hydrophobicity of the ISM and the studied ions, which have opposite negative charges, improved the membrane selectivity. Additionally, MWCNTs were added and proven to be utilized as a transducer layer to enhance sensitivity and increase stability and reproducibility [36]. For the sensing membrane fabrication, PVC was used as a polymer for firmness, and *o*-NPOE was used as a plasticizer, along with THF. The potential profiles of both FLU electrodes are shown in Fig. 2(a). Notably, there was a slight improvement in the response time with GCE sensor but a significant increase in the stability from 30 to 60 days, as shown in Table 1(a).

**Table 1:** (**a**) Electrochemical response properties of the proposed sensors for FLU detection

Parameter	Conventional	GCE
Slope ± SD (mV/decade) ^a^	-57.408	-58.211
Intercept (mV)	189.58	182.88
Accuracy (Mean ± SD)	100.70 ± 1.23	100.37 ± 0.66
Linear concentration range (M)	1 × 10^− 5^ to 1 × 10^− 2^	1 × 10^− 5^ to 1 × 10^− 2^
Correlation coefficient	1.000	0.999
Stability(days)	30	60
working pH range	6–10	6–11
Dynamic response(sec.)	15–30	10–12
Recovery % ±SD of calibration	1.50	1.51
Interday RSD% ^b^	1.710	0.860
Intraday RSD% ^c^	1.090	0.190
Variance	1.22	1.20
LOD	6.5 × 10^− 6^	7.0 × 10^− 6^

^a^: Average of three determinations ^b^ Interday precision (average of three different concentrations of three replicates each (*n* = 9) repeated on three days successively). ^c^ Intraday precision (average of three different concentrations of three replicates each (*n* = 9) within the same day)

For OXY, MWCNTs were proven to be useful as a transducer layer to improve stability [37]. To produce a very sensitive sensor, the feasibility of HP-ßCD as an ionophore on MWCNT-GCE was investigated by comparing the results of this electrode with those of a non-ionophore-modified MWCNT-GCE. Note that the MWCNTs were incorporated into the ISM, an application to increase long-term accuracy and durability, thus increasing selectivity. The most important property of cyclodextrin is its supramolecular chemistry, which aids in equilibrium reactions, leading to inclusion complexes containing organic ions and molecules of appropriate size [38]. Due to the neutral nature of cyclodextrins, they acquire the charge of the guest compound when forming a complex. Multiple ISEs have been described in the literature for the determination of tetracycline, but because of these features, only few of them were found to be beneficial after indirect analysis methods were applied. Further research has offered opportunities to increase the body’s knowledge and thus offers different approaches for enhancing the selectivity and sensitivity of analytes, one of which involves the use of compounds (ionophores) that can selectively extract and sense an analyte’s low activity inside the sensing membrane. In that regard, cyclodextrin was used as an ionophore in the literature in combination with polypyrrole as an ion-electron transducer, with a linearity of 2 × 10^− 5^–10^− 2^ M and a slope of approximately 55 mV/dec. In our study, the linearity range of OXY was extended to 1 × 10^− 6^ M, with a higher slope of 59.47 mV/dec. Figure 2(b) reveals that the potential profile of both OXY electrodes significantly changed by the addition of HP-ßCD; the modified electrode had a greater Nernstian response, slightly shorter dynamic response time, and reproducible potential, as summarized in Table 1(b).

**Table 1:** (**b**) Electrochemical response properties of the proposed sensors for OXY

Parameter	Electrode A	Electrode B
Slope ± SD (mV/decade) ^a^	58.93	59.47
Intercept (mV)	231.39	370.03
Accuracy (Mean ± SD)	100.99 ± 0.81	102.1 ± 1.44
Linear concentration range (M)	1 × 10^− 6^ to 1 × 10^− 2^	1 × 10^− 6^ to 1 × 10^− 2^
Correlation coefficient	1.000	1.000
Stability(days)	60	60
working pH range	2–4	1–5
Dynamic response(sec.)	10–15	10–12
Recovery % ±SD of calibration	1.08	0.38
Interday RSD% ^b^	1.308	0.904
Intraday RSD% ^c^	0.806	0.646
Variance	1.03	0.62
LOD	2.5 × 10^− 7^	3.0 × 10^− 7^

^a^: Average of three determinations ^b^ Interday precision (average of three different concentrations of three replicates each (*n* = 9) repeated on three days successively). ^c^ Intraday precision (average of three different concentrations of three replicates each (*n* = 9) within the same day)

**Fig. 3:**
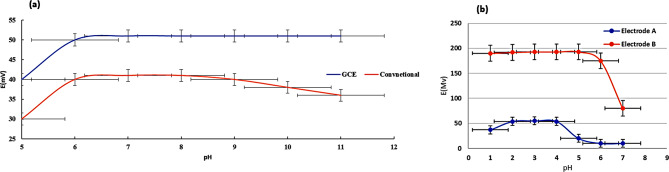
(**a**) Effect of pH on the EMF of Electrode 1 (conventional) and Electrode 2 (MWCNT-GCE) for the determination of FLU. (**b**) Effect of pH on the EMF of Electrode A (GCE) and Electrode B (GCE-HPβ-CD) for the determination of OXY

For the study of pH, the pH working range for FLU was wider with a GCE (6–11) than with a conventional ISE (6–9), as shown in Fig. 3(a).

For OXY, Fig. 3(b) shows that a wider pH working range was observed when using the modified GCE, with a pH range of 1–5 instead of 2–4 when using the unmodified electrode.

**Fig. 4:**
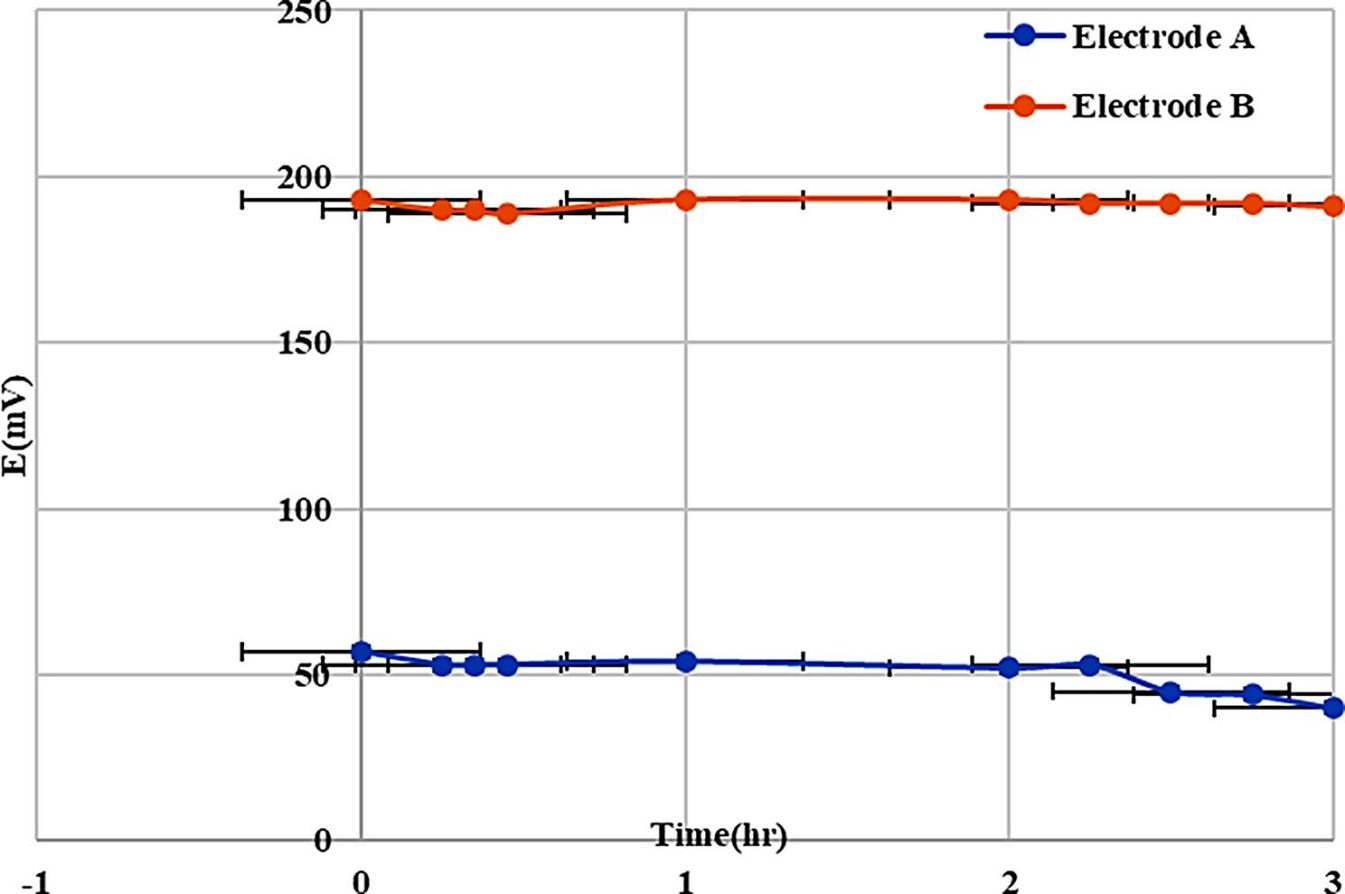
Potential stability measured in 10^−3^ OXY for Electrode A (GCE electrode) and Electrode B (GCE- HP-ßCD) electrode

Moreover, Fig. 4 shows that the modification of the GCE with HP-ßCD had a more stable potential than the unmodified electrode, with a ± 1 mV difference occurring after 3 h in the modified electrode, while there was a ± 3 mV difference in the unmodified electrode.

### Dynamic response time

A major factor for ion-selective electrode applications is the dynamic response time. Regarding FLU electrodes, there was a significant decrease in response time when a GCE was used; the response time was 10–12 s compared to that of the conventional ISE, which was 15 to 30 s, as shown in Table 1(a). This may be attributed to the hydrophobicity and high capacitance of MWCNTs [39].

Regarding OXY, the dynamic response time was slightly decreased by the HP-ßCD-MWCNT-GCE to 10–12 s compared with 10–15 s for the unmodified GCE, as shown in Table 1(b).

This phenomenon may be ascribed to the following factors. The effective surface area of the modified electrode increased as the MWCNTs were modified, allowing additional HP-ßCD to polymerize on the surface of the electrode. In addition, the good electrical conductivity of the MWCNTs improved the conductivity of the electrodes and promoted electron transfer between the electrode and analytes [40].

### Method validation

The proposed method was assessed using ICH guidelines and showed satisfactory results.

#### Linearity

The linearity of the methods was tested by constructing different calibration graphs. The calibration graphs were constructed within the concentration ranges that were selected on the basis of the anticipated drug concentrations. The concentration ranges of FLU and OXY (2.5 × 10^− 4^, 2.5 × 10^− 3^, 1 × 10^− 2^) were tested three times (*n* = 9), which represented the linearity range.

#### Limit of detection (LOD)

The limit of detection (LOD) was calculated from the intersection of the two extrapolated parts of the curve, where it was found to be 6.5 × 10 ^− 6^ and 7.0 × 10 ^− 6^ for Flu electrodes, and 2.5  × 10 ^− 7^ and 3.0 × 10 ^− 7^ for OXY electrodes.

#### Precision

Repeatability and intermediate precision were determined by analyzing three different concentrations of the proposed drugs within the linear range, three times for three pure samples of the drugs on a single day and on three consecutive days, and the results are expressed as RSD%.

##### Repeatability

Repeatability was evaluated by assaying FLU concentrations of 1 × 10^− 3^, 1 × 10^− 4^, and 1 × 10^− 5^ and OXY concentrations of 5 × 10^− 4^, 5 × 10^− 3^, and 1 × 10^− 2^ M-1 in triplicates on the same day, and the mean recovery and RSD% were calculated and found to be ≤ 1.09.

##### Intermediate precision

The intermediate precision of the proposed methods was evaluated by assaying FLU concentrations of 1 × 10^− 3^, 1 × 10^− 4^, and 1 × 10^− 2^ and OXY concentrations of 5 × 10^− 4^, 5 × 10^− 3^, and 5 × 10^− 3^ in triplicates for three successive days, after which the mean recovery percentages and RSD% were calculated and found to be ≤ 1.71.

### Electrode selectivity

The selectivity and specificity of the FLU-sensing electrodes were investigated by observing the potential of 10^− 5^ and 10^− 2^ FLU with the same concentrations of the interfering ions NaCl, Calcium and OXY, while OXY-sensing electrodes were investigated by measuring the potential of 10^− 2^ OXY with FLU, NaCl, Calcium and KCl. Selectivity coefficient values (K ^pot^_A, B_) were obtained by the separate solution method [41], in which the potentials were obtained separately for 10^− 5^ and 10^− 2^M FLU and then for 10^− 5^ and 10^− 2^M interferent solution. Similarly, 10^− 2^ M OXY was measured and then 10^− 2^ M interferent solution. The selectivity coefficients were obtained and are shown in Table 2 using the following equation:

1 $${\rm{log}}\,{{\rm{K}}^{{\rm{pot}}}}_{{\rm{A}},{\rm{B}}} = \left( {{{\rm{E}}_{\rm{B}}} - {{\rm{E}}_{\rm{A}}}} \right)/{\rm{S}} + (1 - {{\rm{Z}}_{\rm{A}}})/{{\rm{Z}}_{\rm{B}}}\,{\rm{log}}\,{{\rm{a}}_{\rm{A}}}$$

where K^pot^_A, B_ is the selectivity coefficient, E_A_ and E_B_ are the potentials of the drug and interferent solutions, S is the slope of the calibration plot, a_A_ is the activity of the drug, and Z_A_ and Z_B_ are the charges of the drug and interfering ions, respectively. Satisfactory selectivity and low interference were observed from the suggested electrodes.

**Table 2:** (**a**) selectivity coefficient^a^ of the proposed electrodes for FLU by the separate solution method (SSM)

	Conventional	GCE
OXY	4.1 × 10^− 3 b^ 4.3 × 10 ^− 3 c^	9.1 × 10^− 4 b^ 9.4 × 10^− 4 c^
Chloride	6.1 × 10^− 3 b^ 6.2 × 10^− 3 c^	4.2 × 10^− 3 b^ 4.0 × 10^− 3 c^
Calcium	6.3 × 10 ^− 3 b^ 6.6 × 10 ^− 3 c^	5.5 × 10 ^− 3 b^ 6.0 × 10 ^− 3 c^

^a^ Each value is the average of three determinations

^b^ FLU interferents are in the form of 1 × 10^− 5^ M

^c^ FLU interferents are in the form of 1 × 10^− 2^ M

**Table 2:** (**b**) selectivity coefficient^a^ of the proposed electrodes for OXY determined by the separate solution method (SSM)

	Electrode A	Electrode B
FLU^b^	6.7 × 10^− 3^	1.2 × 10^− 4^
NaCl^b^	9.5 × 10^− 3^	1.2 × 10^− 4^
KCl^b^	8.5 × 10^− 3^	1.8 × 10^− 4^
Calcium^b^	5.5 × 10^− 3^	5.82 × 10^− 3^

^a^ Each value is the average of three determinations

^b^ OXY interferents are in the form of 1 × 10^− 2^ M

### Application to veterinary formulation

The developed methods were conveniently applied for the assay of OXY and FLU in their combined formulation with satisfactory accuracy (98.50 to 100.78) and precision, as presented in Table 3.

**Table 3:** Applications of the proposed sensors in pharmaceutical dosage form (Floxon^®^ injection-batch no. 180362) and in spiked milk

FLU		
	Conventional	GCE
Floxon^®^ Injection Mean ± SD	98.50 ± 1.59	100.78 ± 1.00
Spiked milk Mean ± SD	99.91 ± 0.57	100.21 ± 0.57
**OXY**		
	**Electrode A**	**Electrode B**
Floxon^®^ Injection Mean ± SD	99.71 ± 0.98	99.63 ± 0.363
Spiked milk Mean ± SD	100.17 ± 1.41	100.23 ± 0.82

### Application to spiked milk

The proposed methods were successfully applied for the determination of both drugs in spiked milk samples with acceptable recoveries ranging from 99.21 to 100.23, as shown in Table 3.

### Greenness assessment

Analysts should always consider the surrounding environment and health of other living beings, especially because the analysis routine is an ongoing procedure with everyday applications, so it would be dangerously cruel to the environment if an analyst developed a non-green method. One should always be aware of the GAC rules and try the best to abide by them. Greening an analytical procedure may be accomplished by different strategies, from carefully choosing the number of solvents to reducing the amount of waste. We employed potentiometric analysis to help us limit the number of solvents used in the proposed processes and choose the least harmful solvents. Because assessing greenness is needed, we assessed our method using one of the most common methods, GAPI [42], along with a new, very promising software for green analysis, AGREE [43].

#### Green analytical procedure index (GAPI)

GAPI has a major advantage in that it evaluates greenness beginning from sample collection until determination, enabling a comprehensive overview of the suggested methods. The results include five pentagrams, each containing three levels of scale color that represent each stage. Green represents low environmental impact, yellow represents medium-low environmental impact, and red represents high environmental impact. In this study, the sample preparation step was not performed, so the pentagram was removed. The GAPI pentagram has green dominance, which satisfyingly means that the method has a low hazard. There were two yellow colors, one for using 10–100 mL of solvent and the other for using THF during fabrication; there was only one red color referring to the total NFPA score of all the solvents. The GAPI pentagram is illustrated in Figure S1.

#### Analytical GREEnness metric approach and software (AGREE)

AGREE is a comprehensive and simple assessment method. Among all greenness assessment methods, only AGREE includes all twelve principles of green analytical chemistry. The greenness profile of this study was also assessed using the AGREE calculator, and the predicted AGREE score involving all 12 different concepts of GAC for this study is shown in Figure S2. AGREE analytical scores greater than 0.75 indicate that the analytical method is suitable for routine drug determination. Moreover, a score of 0.50 indicated that the method is fit for routine drug determination. Finally, scores less than 0.50 indicated the unacceptability of the proposed analytical method. The AGREE score for this study’s proposed electrodes was 0.82, which means that the method has excellent greenness and could be safely applied for routine analysis. This study represents the first use of this metric system for potentiometric analysis.

### Comparison of other analytical techniques for analysis of OXY

Numerous potentiometric methods have been published for determining OXY in different matrices and are summarized in Table S1, but none of them have studied the effect of MWCNTs on potential response in milk samples and pharmaceutical formulations.

### Statistical analysis

A statistical comparison of the results obtained for both drugs by the proposed electrodes and the official methods showed no significant differences, as shown in Tables S2, S3 and S4.

## Conclusion

The proposed study describes adopting GAC principles for electrochemical determination of FLU and OXY. This work describes a comparison between two potentiometric electrodes for each drug. Concerning FLU sensors, the inclusion of MWCNTs as an ion-to-electron transducer added valuable stability compared to that of the conventional ISE. On the other hand, for OXY, modification of MWCNT-GCE with HP-ßCD was a promising approach for increasing the electrode stability and selectivity.

The described sensors offer the advantages of fast response, drug pretreatment or separation steps exclusion, good selectivity, low detection limit, and successful drug determination in various matrices. Therefore, the developed sensors are successfully applicable to routine analysis of both OXY and FLU in different matrices.

## Electronic supplementary material

Below is the link to the electronic supplementary material.

Supplementary Material 1

## Data Availability

Data is provided within the manuscript or supplementary information files.

## References

[1] Aminov RI. A brief history of the antibiotic era: lessons learned and challenges for the future. Front Microbiol. 2010;1:134. 10.3389/FMICB.2010.00134/BIBTEX. 21687759 10.3389/fmicb.2010.00134 PMC3109405

[2] Prestinaci F, Pezzotti P, Pantosti A. Antimicrobial resistance: a global multifaceted phenomenon. Http://Dx Doi Org. 2015;109309–18. 10.1179/2047773215Y.0000000030. 10.1179/2047773215Y.0000000030 PMC4768623 26343252

[3] Sachi S, Ferdous J, Sikder MH, Azizul Karim SM, Hussani. Antibiotic residues in milk: past, present, and future. J Adv Vet Anim Res. 2019;6:315–32. 10.5455/JAVAR.2019.F350. 31583228 10.5455/javar.2019.f350 PMC6760505

[4] Hassan HF, Saidy L, Haddad R, Hosri C, Asmar S, Jammoul A, Jammoul R, Hassan H, Serhan M. Investigation of the effects of some processing conditions on the fate of oxytetracycline and tylosin antibiotics in the making of commonly consumed cheeses from the East Mediterranean. Vet World. 2021;14:1644. 10.14202/VETWORLD.2021.1644-1649. 34316215 10.14202/vetworld.2021.1644-1649 PMC8304414

[5] Borriello S, Broadfoot P, Healey F, Neale K, Pickering D, Sabzikari A. UK Veterinary Antibiotic Resistance and Sales Surveillance Report, 2020.

[6] Ayad MF, Trabik YA, Abdelrahman MH, Fares NV, Magdy N. Potentiometric carbon quantum dots-based screen-printed arrays for nanotracing gemifloxacin as a model fluoroquinolone implicated in antimicrobial resistance. Chemosensors. 2021;9:1–16. 10.3390/chemosensors9010008.

[7] Covington AK. Ion selective electrode method, CRC Press, 2018. 10.1201/9781351073875/ION-SELECTIVE-ELECTRODE-METHODOLOGY-COVINGTON-ARTHUR.

[8] Zhu C, Yang G, Li H, Du D, Lin Y. Electrochemical Sensors and biosensors based on nanomaterials and nanostructures. Anal Chem. 2014;87:230–49. 10.1021/AC5039863. 25354297 10.1021/ac5039863 PMC4287168

[9] Elghobashy MR, Mahmoud AM, Rezk MR, Abd El-Rahman MK. Strategy for fabrication of stable tramadol solid-contact ion-selective Potentiometric Sensor based on Polyaniline nanoparticles. J Electrochem Soc. 2015;162:H1–5. 10.1149/2.0161501JES/XML.

[10] Trabik YA, Al-Afify NKH, El-Kosasy AM, Magdy N. Application of precipitation-based and nanoparticle-based Techniques for Fabrication of Potentiometric Sensors for Nano Molar Determination of Chitosan and polyvinyl pyrrolidone in Pharmaceutical formulations and Biological fluids. Electroanalysis. 2021;33:1233–43. 10.1002/ELAN.202060492.

[11] Zhou M, Gan S, Cai B, Li F, Ma W, Han D, Niu L. Effective solid contact for ion-selective electrodes: Tetrakis(4- chlorophenyl)borate (TB -) anions doped nanocluster films. Anal Chem. 2012;84:3480–3. 10.1021/AC300473A/SUPPL_FILE/AC300473A_SI_001.PDF. 22409283 10.1021/ac300473a

[12] Crespo GA, Macho S, Bobacka J, Rius FX. Transduction Mechanism of Carbon Nanotubes in Solid-Contact Ion-Selective electrodes. Anal Chem. 2008;81:676–81. 10.1021/AC802078Z. 10.1021/ac802078z 19093752

[13] Hareesha N, Manjunatha JG. Electro-oxidation of formoterol fumarate on the surface of novel poly (thiazole yellow-G) layered multi-walled carbon nanotube paste electrode. Sci Rep. 2021;11(1):12797. 34140565 10.1038/s41598-021-92099-x PMC8211837

[14] Mahmoud AM, Abd El-Rahman MK, Elghobashy MR, Rezk MR. Carbon nanotubes versus polyaniline nanoparticles; which transducer offers more opportunities for designing a stable solid contact ion-selective electrode. J Electroanal Chem. 2015;755:122–6. 10.1016/J.JELECHEM.2015.07.045.

[15] Ogoshi T, Harada A. Chemical Sensors Based on Cyclodextrin Derivatives, Sensors 2008, Vol. 8, Pages 4961–4982. 8 (2008) 4961–4982. 10.3390/S8084961 10.3390/s8084961 PMC3705481 27873795

[16] El-Kosasy AM, abd El Aziz L, Trabik YA. Comparative study of Beta cyclodextrin and Calix-8-Arene as ionophores in Potentiometric Ion-Selective electrodes for Sitagliptin phosphate. J Appl Pharm Sci. 1AD;2:51–6. 10.7324/JAPS.2012.2806.

[17] The United States Pharmacopeial Convention. United State Pharmacopoeia USP. 2011;35:867–8.

[18] Fouad MM, Abd El-Razeq SA, Belal FF, Fouad FA. Spectrophotometric methods for the determination of flunixin meglumine and menbutone in bulk and dosage forms. Int J Pharm Anal. 2013;4:30.

[19] Belal FF, Abd El-Razeq SA, Fouad MM, Fouad FA. Micellar high-performance liquid chromatographic determination of flunixin meglumine in bulk, pharmaceutical dosage forms, bovine liver and kidney. Anal Chem Res. 2015;3:63–9. 10.1016/J.ANCR.2014.12.003.

[20] Zhang Q, Mi S, Xie Y, Yu H, Guo Y, Yao W. Core-shell Au@ MIL-100 (fe) as an enhanced substrate for flunixin meglumine ultra-sensitive detection. Spectrochim Acta Part A Mol Biomol Spectrosc. 2023;287:122018. 10.1016/j.saa.2022.122018 36332394

[21] Radi AE, Abd El-Ghany N, Wahdan T. Voltammetric determination of Flunixin on Molecularly Imprinted Polypyrrole Modified Glassy Carbon Electrode. J Anal Methods Chem. 2016;2016. 10.1155/2016/5296582. 10.1155/2016/5296582 PMC4876014 27242945

[22] Ivković B, Zabelaj D, Brborić J, Čudina. O. Development and validation of high-performance liquid chromatography method for determination of flunixin-meglumine and its impurities in preparations for veterinary use. Archives Pharm. 2022;72:S540–1.

[23] Lehotay SJ, Le Floch M, Lightfield AR, Couëdor P, Hurtaud-Pessel D, Michlig N. Stability study of selected veterinary drug residues spiked into extracts from different food commodities. Food Addit Contaminants: Part A. 2023;40(9):1198–217. 10.1080/19440049.2023.2240444 37582153

[24] Park D, Choi YS, Kim JY, Choi JD, Moon GI. Determination of Flunixin and 5-Hydroxy flunixin residues in livestock and Fishery products using Liquid Chromatography-Tandem Mass Spectrometry (LC-MS/MS). Food Sci Anim Resour. 2024;44(4):873. 38974729 10.5851/kosfa.2024.e24 PMC11222691

[25] Meucci V, Vanni M, Sgorbini M, Odore R, Minunni M, Intorre L. Determination of phenylbutazone and flunixin meglumine in equine plasma by electrochemical-based sensing coupled to selective extraction with molecularly imprinted polymers. Sens Actuators B. 2013;179:226–31.

[26] Toral MI, Sabay T, Richter P, Boison J, Turnipseed S. Determination of Oxytetracycline from Salmon Muscle and skin by Derivative Spectrophotometry Methods of Analysis for Residues and Chemical contaminants in Aquaculture. J AoaC Int. 2015;98:559. 10.5740/jaoacint.14-027. 26025109 10.5740/jaoacint.14-027

[27] Gajda A, Jablonski A, Bladek T, Posyniak A. Oral fluid as a biological material for antemortem detection of oxytetracycline in pigs by liquid chromatography- Tandem mass spectrometry. J Agric Food Chem. 2017;65:494–500. 10.1021/acs.jafc.6b05205. 28042939 10.1021/acs.jafc.6b05205

[28] Gil RL, Amorim CMPG, Conceição M, Montenegro BSM, Araújo AN. Cucurbit [8] uril-based potentiometric sensor coupled to HPLC for determination of tetracycline residues in milk samples. Chemosensors. 2022;10(3):98.

[29] Ghodsi J, Rafati AA, Shoja Y. First report on electrocatalytic oxidation of oxytetracycline by horseradish peroxidase: application in developing a biosensor to oxytetracycline determination. Sens Actuators B Chem 224 (2016) 692–9. 10.1016/J.SNB.2015.10.091

[30] Nazlawy HN, Zaazaa HE, Merey HA, Atty SA. Green validated chromatographic methods for simultaneous determination of co-formulated oxytetracycline HCl and flunixin meglumine in the presence of their impurities in different matrices. J Iran Chem Soc. 2023;20(4):885–96.

[31] International Conference on Harmonization, Guideline for Elemental Impurities Q3D (R1). John Wiley & Sons, Inc., 2019. 10.1002/9781118971147

[32] Monir HH, Fayez YM, Nessim CK, Michael AM. When is it safe to eat different broiler chicken tissues after administration of doxycycline and tylosin mixture? J Food Sci. 2021;86:1162–71. 10.1111/1750-3841.15640. 33598923 10.1111/1750-3841.15640

[33] Shao Y, Ying Y, Ping J. Recent advances in solid-contact ion-selective electrodes: functional materials, transduction mechanisms, and development trends. Chem Soc Rev. 2020;49:4405–65. 10.1039/C9CS00587K. 32458836 10.1039/c9cs00587k

[34] Tajik S, Dourandish Z, Nejad FG, Beitollahi H, Jahani PM, Bartolomeo AD. Transition metal dichalcogenides: synthesis and use in the development of electrochemical sensors and biosensors. Biosens Bioelectron. 2022;216:114674. 36095980 10.1016/j.bios.2022.114674

[35] Mahmoud AM, Moaaz EM, Rezk MR, Abdel-Moety EM, Fayed AS. Microfabricated solid-contact Potentiometric Sensor for Determination of Tedizolid Phosphate, application to Content Uniformity Testing. Electroanalysis. 2022. 10.1002/elan.202200115.

[36] Fares MY, Abdelwahab NS, Hegazy MA, Abdelrahman MM, Mahmoud AM, EL-Sayed GM. Nanoparticle-enhanced in-line potentiometric ion sensor for point-of-care diagnostics for tropicamide abuse in biological fluid. Anal Chim Acta. 2022;1192:339350. 10.1016/j.aca.2021.339350. 35057968 10.1016/j.aca.2021.339350

[37] Wu K, Fei J, Hu S. Simultaneous determination of dopamine and serotonin on a glassy carbon electrode coated with a film of carbon nanotubes. Anal Biochem. 2003;318:100–6. 10.1016/S0003-2697(03)00174-X. 12782037 10.1016/s0003-2697(03)00174-x

[38] Hussein LA, El-Kosasy AM, Trabik YA. Comparative study of normal, micro- & nanosized iron oxide effect in potentiometric determination of fluconazole in biological fluids. RSC Adv. 2015;5:37957–63. 10.1039/C5RA05245A.

[39] Trabik YA, Ismail RA, Ayad MF, Hussein LA, Mahmoud AM. Microfabricated potentiometric sensor based on a carbon nanotube transducer layer for selective Bosentan determination. Rev Anal Chem. 2024;43(1):20230071.

[40] Li Y, et al. Nonenzymatic sensing of uric acid using a carbon nanotube ionic-liquid paste electrode modified with poly (β-cyclodextrin). Microchim Acta. 2015;182:1877–84.

[41] Umezawa Y, Bühlmann P, Umezawa K, Tohda K, Amemiya S. Potentiometric selectivity coefficients of ion-selective electrodes part I. Inorganic cations (technical report), Pure Appl. Chem. 72 (2000) 1851–2082. 10.1351/pac200072101851

[42] Płotka-Wasylka J. A new tool for the evaluation of the analytical procedure: Green Analytical Procedure Index. Talanta. 2018;181:204–9. 10.1016/J.TALANTA.2018.01.013. 29426502 10.1016/j.talanta.2018.01.013

[43] Pena-Pereira F, Wojnowski W, Tobiszewski M. AGREE - Analytical GREEnness Metric Approach and Software. Anal Chem. 2020;92:10076–82. 10.1021/acs.analchem.0c01887. 32538619 10.1021/acs.analchem.0c01887 PMC7588019

