# Supplementary material for: Eco-friendly electrochemical assay of oxytetracycline and flunixin in their veterinary injections and spiked milk samples
Source: BMC Chem. 2024 Sep 19;18(1):179. doi: 10.1186/s13065-024-01282-4 (PMC11411790; doi:10.1186/s13065-024-01282-4)
Supplement: Supplementary file 1 — Supplementary Material 1 [file 13065_2024_1282_MOESM1_ESM.docx]

**Supplementary Material**

**Table S1.** comparison of other published potentiometric techniques for analysis of OXY

| Technique | Matrix |  | Linear range (M) | LOD (M) | Reference | |
| --- | --- | --- | --- | --- | --- | --- |
| Potentiometry | Spiked milk samlpes |  | 1.0 × 10^−10^ to 1.0 × 10^−5^ | 2.3 × 10^−11^ | | 1 |
| Potentiometry | Water analysis |  | 4.0 × 10^–7^–5.0 × 10^–2^ | (4.3±0.2) ×10^−5^ | | 2 |
| Potentiometry | pharmaceutical formulation |  | 2.0×10^−5^–10^−2^ | 1.0 × 10^–7^ | | 3 |

**Table S2.** Statistical comparison of the results obtained by the proposed sensors with those obtained by the reported method for the analysis of FLU

|  | **USP method^a^** | **Conventional** | **GCE** |
| --- | --- | --- | --- |
| **Mean** | 100.08 | 100.02 | 100.50 |
| **SD** | 1.26 | 1.49 | 1.50 |
| **Variance** | 1.12 | 1.22 | 1.09 |
| **N** | 6 | 6 | 6 |
| **Student’s t test (2.228)** | ----- | 0.08 | 0.361 |
| **F Value**  **(5.05)** | ----- | 1.09 | 1.10 |

^a^ Titrimetric method.

**Table S3.** Statistical comparison of the results obtained by the proposed sensors with those obtained by the reported method for the analysis of OXY

|  | **USP method ^a^** | **Electrode A** | **Electrode B** |
| --- | --- | --- | --- |
| **Mean** | 99.48 | 100.03 | 99.12 |
| **SD** | 1.43 | 1.88 | 0.38 |
| **Variance** | 1.19 | 1.37 | 0.62 |
| **N** | 6 | 6 | 6 |
| **Student’s t test (2.228)** | ----- | 0.358 | 0.128 |
| **F Value**  **(5.05)** | ----- | 1.15 | 1.94 |

^a^ Direct spectrophotometric determination at 353 nm.

**Table S4.** One way ANOVA was used for the proposed methods, and the USP official method for the determination of FLU and OXY in pure powder form:

| FLU | | | | | | |
| --- | --- | --- | --- | --- | --- | --- |
| Source of Variation | SS | df | MS | F | P value | F crit |
| Between Groups | 12.01492 | 2 | 6.007462 | 2.952388 | 0.128024 | 5.143253 |
| Within Groups | 12.20869 | 6 | 2.034781 |  |  |  |
| Total | 24.22361 | 8 |  |  |  |  |
| OXY | | | | | | |
| Source of Variation | SS | df | MS | F | P value | F crit |
| Between Groups | 6.981101 | 2 | 3.49055 | 3.410248 | 0.102504 | 5.143253 |
| Within Groups | 6.141284 | 6 | 1.023547 |  |  |  |
| Total | 13.12238 | 8 |  |  |  |  |


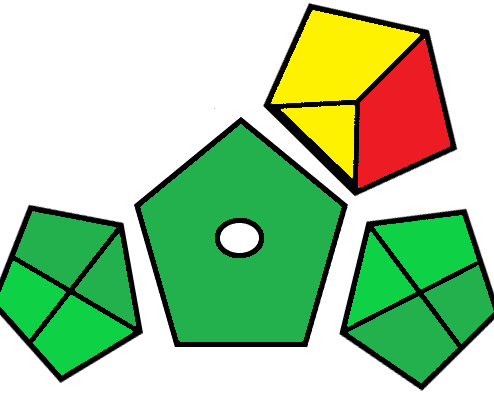


**Figure S1.** GAPI green profile assessment of the proposed methods.


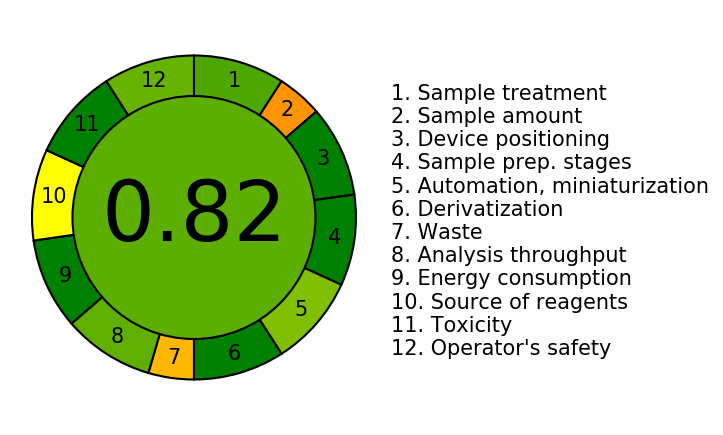


**Figure S2.** Results of AGREE green profile assessment software.

References

1. Mliki, H., Rouis, A., Echabaane, M., Ceron, L., Perol, N., Darbost, U., Bonnamour, I., Bessueille, F., Ayed, D. and Jaffrezic-Renault, N., 2024. A highly sensitive and selective impedimetric sensor for the determination of oxytetracycline based on a new polyamine functionalized calix [4] arene. *Microchemical Journal*, *199*, p.109957.

2. Cunha, Cláudia O., Rita CR Silva, Célia G. Amorim, Severino A. Júnior, Alberto N. Araújo, Maria CBSM Montenegro, and Valdinete L. Silva. "Tetracycline potentiometric sensor based on cyclodextrin for pharmaceuticals and wastewater analysis." *Electroanalysis* 22, no. 24 (2010): 2967-2972.

3.Sun, Xian Xiang, Xu Zhang, and Hassan Y. Aboul-Enein. "Construction and characterization of potentiometric sensor for the determination of oxytetracycline hydrochloride." *Il Farmaco* 59.4 (2004): 307-314.
